# Transcriptional Defect of an Inherited NKX2-5 Haplotype Comprising a SNP, a Nonsynonymous and a Synonymous Mutation, Associated with Human Congenital Heart Disease

**DOI:** 10.1371/journal.pone.0083295

**Published:** 2013-12-20

**Authors:** Stella Marie Reamon-Buettner, Evelyn Sattlegger, Yari Ciribilli, Alberto Inga, Armin Wessel, Jürgen Borlak

**Affiliations:** 1 Fraunhofer Institute of Toxicology and Experimental Medicine, Hannover, Germany; 2 Institute of Natural Sciences, Massey University, Auckland, New Zealand; 3 Laboratory of Transcriptional Networks, Centre for Integrative Biology, University of Trento, Mattarello, Italy; 4 Department of Pediatric Cardiology and Intensive Care Medicine, Hannover Medical School, Hannover, Germany; 5 Centre for Pharmacology and Toxicology, Hannover Medical School, Hannover, Germany; New York Medical College, United States of America

## Abstract

Germline mutations in cardiac-specific transcription factor genes have been associated with congenital heart disease (CHD) and the homeodomain transcription factor NKX2-5 is an important member of this group. Indeed, more than 40 heterozygous NKX2-5 germline mutations have been observed in individuals with CHD, and these are spread along the coding region, with many shown to impact protein function. In pursuit of understanding causes of CHD, we analyzed n = 49 cardiac biopsies from 28 patients and identified by direct sequencing two nonsynonymous *NKX2-5* alterations affecting alanine 119, namely c.356C>A (p.A119E) and c.355G>T, (p.A119S), in patients with AVSD and HLHS, respectively. In functional assays, a significant reduction in transcriptional activities could be determined for the *NKX2-5* variants. Importantly, in one family the mother, besides p.A119E, carried a synonymous mutant allele in the homeodomain (c.543G>A, p.Q181), and a synonymous dbSNP (c.63A>G, p.E21) in the transactivation domain of the protein, that were transmitted to the CHD daughter. The presence of these variants in*-cis* with the p.A119E mutation led to a further reduction in transcriptional activities. Such difference in activity may be in part related to reduced protein expression for the double variant c.356C>A and c.543G>A. We propose changes in mRNA stability and folding, due to a silent mutation and a dbSNP in the *NKX2-5* coding region to contribute to the functional defect. Although the clinical significance of the NKX2-5 haplotype identified in the CHD patients remains to be ascertained, we provide evidence of an interaction of a dbSNP, with synonymous and nonsynonymous mutations to negatively impact NKX2-5 transcriptional activity.

## Introduction

Deciphering the exact causes of congenital heart disease (CHD) in humans is a complicated task. First of all, many patients do not have familial background of the disease. Although germline mutations in several transcription factor genes that govern heart development have been implicated in the disease, detection frequency is low. In sporadic case of CHD, detection frequency, for instance, for *NKX2-5* (NK2 transcription factor related, locus 5) ranged from 0 to about 3% [1]–[3]. More than 40 different *NKX2-5* mutations have been identified so far, but only five were detected more than once in unrelated individuals. Thus, families have their own ‘private’ mutation, and such mutation can lead to a variety of cardiac defects even within the same family. This suggests that CHD cannot just be explained by simple monogenic inheritance or by a single germline mutation. Indeed, there is emerging evidence that CHD is a multifactorial disease in which genetic factors, environmental factors and gene-environment interactions are key events resulting in mutations, chromosomal aberrations or abnormal gene expression (see reviews [4], [5]).

Our work on the Leipzig collection of malformed hearts suggests that somatic mutations in cardiac-specific transcription factor genes may have a role in CHD. For instance, we showed recently that mutations in *HAND1* (heart and neural crest derivatives expressed 1) may contribute to hypoplasia or to septation defects of the human hearts [6], [7]. The cause for these somatic mutations is unknown to us, but gene-environment interactions might be a trigger. In this paper, we report the genetic analysis on discarded cardiac biopsies of CHD patients undergoing heart surgery. Overall, we examined 49 biopsies from 28 patients with various heart malformations, including septal defects, Tetralogy of Fallot (TOF), and hypoplastic left heart syndrome (HLHS). Direct sequencing of *NKX2-5* and *HAND1* revealed a total of three heterozygous nonsynonymous sequence alterations, two in *NKX2-5* and one in *HAND1*. Both *NKX2-5* sequence alterations identified in patients of different cardiac disease phenotypes affected the alanine residue at position 119 and were passed on from an unaffected parent.

## Materials and Methods

### Ethics statement

Recruitment of patients and handling of samples was according to an approved protocol from the Ethics Committee, Hannover Medical School. The ethical vote was obtained by Prof. Armin Wessel. Participants provided their written informed consent to participate in this study. Blood and tissue samples were obtained from the Department of Pediatric Cardiology of patients undergoing cardiac surgery for diverse cardiac malformations at the Hannover Medical School, Germany. The ethics committees approved the procedure which was documented in the study protocol.

### Blood and heart tissues

Heart tissues came from discarded biopsies as a result of surgery; thus varying in size, number and not necessarily within the malformation itself.

### Genomic DNA isolation, mutation analysis

Genomic DNA was isolated with NucleoSpin Tissue or Blood Kit (Macherey-Nagel, Dueren, Germany). A typical PCR reaction consisted of 20–50 ng of genomic DNA, 1x PCR buffer, 1 U of Hot Star *Taq* DNA polymerase (Qiagen, Hilden, Germany), 5 µl of 5x Q-Solution (Qiagen), 0.2 mM dNTPs, 5 pmol of each primer pair, to a volume of 25 µl with distilled water. After an initial 15 min activation step at 95°C, a typical PCR program included 35 cycles of 10 s at 94°C denaturation, 30 s at 60°C annealing, and 2 min at 68°C, elongation; a final extension of 10 min, 68°C followed by indefinite 4°C. PCR reactions were carried out on Biometra thermocyclers (Biometra, Goettingen, Germany).

PCR-amplified fragments were sequenced directly in both directions using BigDyeTerminator v3.1 Kit (Applied Biosystems, Darmstadt, Germany) and Applied Biosystems 3100 Genetic Analyzer. Sequences were analyzed using SeqScape 2.0 (Applied Biosystems) or DNASTAR Lasergene 7.0 (Madison, Wisconsin, USA). The numbering of sequence alterations within the coding region of the gene starts with the nucleotide A of the first codon. Unless reported as NCBI dbSNPs (NCBI database Single Nucleotide Polymorphism), we refer to nucleotide changes as sequence variations or mutations interchangeably, which simply mean deviations from the reference sequence, e.g. for *NKX2-5* (NM_004387.2), for *HAND1* (NM_004821), that may or may not be disease-causing.

### Bioinformatic prediction of protein function and prediction of RNA secondary structure

For prediction of the functional consequences of mutations on protein sequence, we used three software programs available in the internet, namely: PMut (http://mmb2.pcb.ub.es:8080/PMut/), PolyPhen (http://genetics.bwh.harvard.edu/pph/) and SIFT (http://sift.jcvi.org/). For prediction on mRNA secondary structure, we used GeneQuest (DNASTAR Lasergene 7.0), which uses the Vienna RNA folding procedure, taken from Zuker's optimal RNA folding algorithm, to fold the sense strand of selected DNA regions as RNA.

### Yeast-based luciferase assays

Gene reporter assays in yeast were developed using the available NKX2-5 reporter strains and following the protocol previously described [8]. Briefly, *NKX2-5* sequence variants were constructed into the expression vectors using PCR-based site-directed mutagenesis followed by gap repair. The *NKX2-5* expression vectors were transformed into the yeast reporter strains using a standard LiAc protocol. Transformants were isolated and purified exploiting the *TRP1* selection marker on the plasmid and cultured for 24 hours in selective liquid medium containing variable amount of galactose, to achieve variable induction of NKX2-5 proteins under the *GAL1,10* promoter. Cells were then collected by centrifugation and lysed using glass beads and a commercial lysis buffer (Promega, Milan, Italy). Luciferase activity was quantified using a luminometer and normalized to the amount of soluble proteins. Results were confirmed by independent experiments carried out in different labs, i.e. AI (Italy), JB (Germany) and ES (New Zealand).

### Western blot analysis

Yeast whole cell extracts were resolved in 4–12% SDS-PAGE gradient gels, and the proteins transferred to a nitrocellulose membrane (Invitrogen, Milan, Italy). Proteins were subjected to immunoblotting using mouse antibodies against the TP53 TAD domain (sc-126, Santa Cruz, Biotechnology, USA) that is present as amino terminal fusion to the entire NKX2-5 coding sequence in the yeast expression vectors and against yeast 3-phosphoglycerate kinase (PGK1, Invitrogen). Immune complexes were visualized using horseradish peroxidase conjugated to anti-mouse antibodies (Sigma-Aldrich, Milan, Italy), and the Biorad ChemiDocXRS+ digital imaging equipment (Biorad, Milan, Italy).

## Results

### Sequence analysis identified *NKX2-5* and *HAND1* mutations

**Figure 1:**
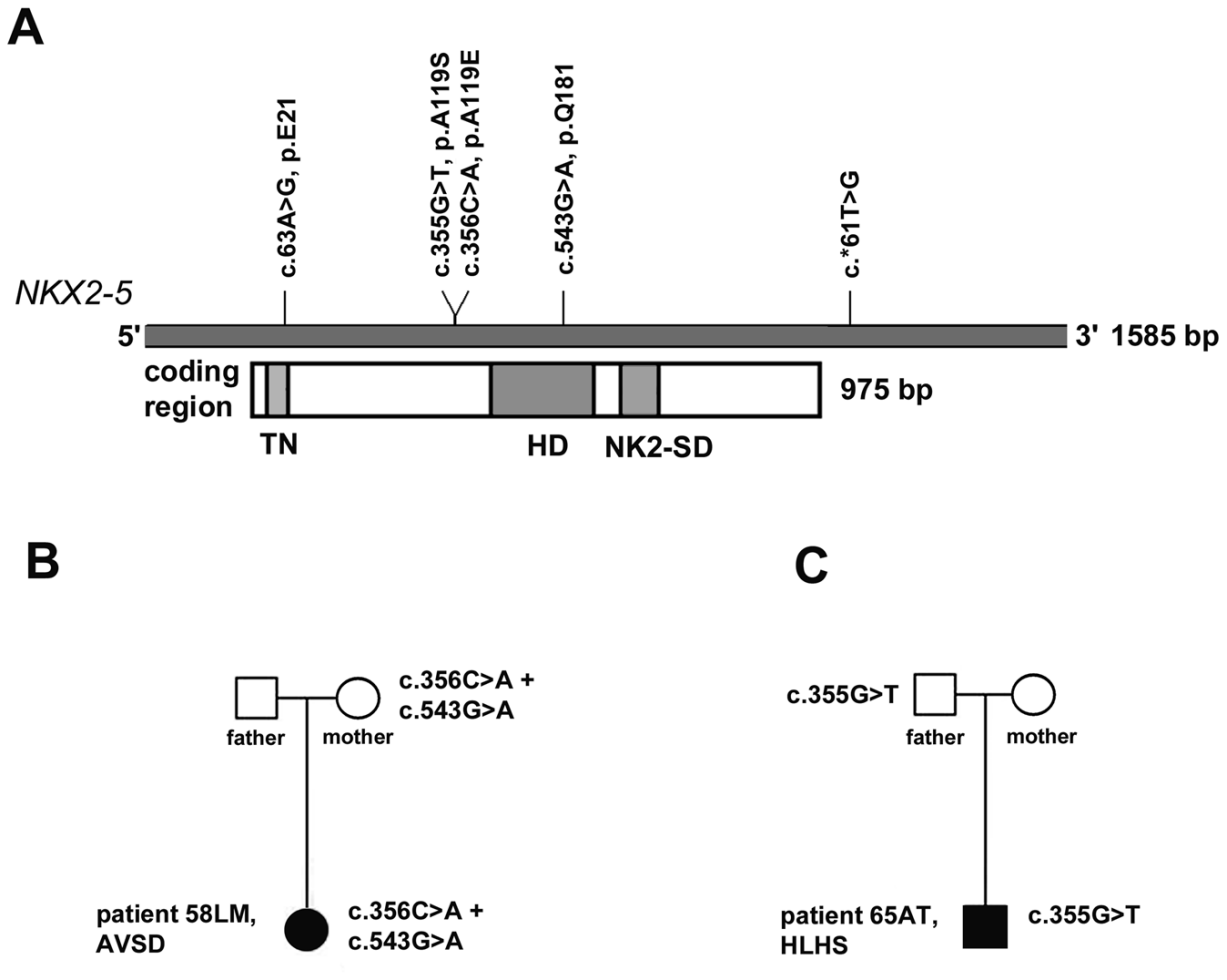
Summary of identified *NKX2-5* sequence variations in patients with CHD. (**A**) their location along the gene; (**B, C**) patients and parents positive for the mutation.

We investigated genomic DNA isolated from 49 heart tissues of 28 Caucasian patients with various heart abnormalities, including septal defects as well as TOF and HLHS (Table 1). The heart tissues were discarded biopsies from patients undergoing cardiac surgery, very minute for obvious reasons, and mostly not within the malformation itself. Search for mutations in *NKX2-5*, identified two patients who were heterozygous for three sequence alterations including two that will affect the alanine residue at position 119, i.e. c.356C>A (p.A119E) and c.355G>T, (p.A119S). The third was located in the homeodomain of NKX2-5, but will not lead to an amino acid change (c.543G>A, p.Q181, synonymous mutation). No other sequence alterations were observed, except the dbSNPs rs2277923 (c.63A>G, p.E21; 10AA:15AG:3GG) and rs703752 (c.*61T>G, 3TT:13TG:12GG). The location of these sequence alterations and the dbSNPs along the *NKX2-5* gene is depicted in Fig. 1A.

**Table 1:** Cardiac malformations of patients, biopsies for mutation analysis, and *NKX2-5* and *HAND1* variations.

Patient ID	Cardiac malformations and other anomalies	No.of tissue	Source of tissue*	*NKX2-5*			*HAND1*
					rs2277923	rs703752	
26SS	aortic stenosis	2	RA, aortic valve + aorta ascendens		R	K	
30KK	VSD, Down syndrome	1	RA		R	K	
34JS	AVSD, Down syndrome	1	RA		R	G	
39YO	HLHS	2	RA near septum, myocardium RA		R	G	
44KB	subaortic VSD, double-chambered RV	3	RA, infundibulum, myocardium RV		R	K	
49NS	HLHS	1	RA		G	G	
52SP	HLHS	1	RA		A	K	
58LM	AVSD	2	RA, myocardium LV with papillary muscle	c.356C>A + c.543G>A	R	K	
61MC	TOF	3	RA, myocadium RV, infundibulum		R	K	
65AT	HLHS	1	RA	c.355G>T	R	K	
69MB	perimembranous VSD	1	RA		R	G	
72DR	TOF, Down syndrome	1	myocardium RV		G	G	
80HO	ASD sinus venosus, TAPVR	2	RA, LV		A	G	
89RR	VSD	2	RA, conduit RV-PA		A	K	
93RH	HLHS	1	RA		R	K	
96JF	VSD, aortic isthmus stenosis, aortic stenosis	1	aortic valve		R	G	
99NM	ASD	1	RA		A	T	c.252G>T
103JR	mitral valve stenosis, persistent left superior vena cava	3	RA, mitral valve, papillary muscle		A	G	
107CC	DORV, AVSD, hypoplasia of left AV valve and LV	2	RA, atrial septum		G	G	
111MO	aortic isthmus stenosis, aortic stenosis	2	RA, aortic valve		R	K	
114CL	AVSD, TOF	1	RVOT muscle		A	K	
117HT	VSD, TGA, pulmonary stenosis	2	RA, myocardium RV		A	G	
120DZ	hypertophy, cardiomyopathy	2	RA, myocardium and septum LV		A	G	
124MS	VSD	1	RA		A	T	
127PB	truncus cummunis, diGeorge	3	RA, myocardium RV, truncus		R	K	
130KM	tricuspid atresia	2	RA		R	G	
134MS	TOF	2	RA, RVOT infundibulum		R	K	
138LV	subaortic stenosis, aortic isthmus stenosis	3	RA, LVOT fibrous membrane		A	T	

* RA  =  right atrium, VSD  =  ventricular septal defect, RV  =  right ventricle, LV  =  left ventricle, PA  =  pulmonary artery, RVOT  =  right ventricular outflow tract, RVOT  =  left ventricular outflow tract.

Patient #58LM diagnosed with atrioventricular septal defect (AVSD) had c.356C>A (p.A119E) and the synonymous mutation c.543G>A (p.Q181). Patient #65AT diagnosed with hypoplastic left heart syndrome (HLHS) had c.355G>T (p.A119S). These three sequence alterations were also present in blood samples of the same patients as well in one of their parents, evidencing their germline origin (Fig. 1B, C). Both patients came from unaffected families. Their parents carried the sequence alterations but were disease-free. Moreover, patient #58LM and the mother were heterozygous for c.63A>G (dbSNP rs2277923), c.356C>A (p.A119E), c. 543G>A (p.Q181) and c.*61T>G (dbSNP rs703752). To determine the haplotypes in the patient, we used a pair of PCR primers to amplify the entire *NKX2-5* gene, i.e. encompassing the two exons and intervening intronic sequences. Sequencing of clones found two haplotypes, i.e. haplotype 1- with all reference alleles [c.63A; c.356C; c.543G; c.*61T], and haplotype 2 - all variant alleles [c.63G; c.356A; c.543A; c.*61G]. The same haplotypes were obtained with the mother's DNA, suggesting that the variants were transmitted concomitantly.

We also analyzed the 49 heart tissues of 28 patients for *HAND1* mutations and detected a nonsynonymous mutation (c.252G>T, p.R84L) in the right atrium of an atrial septal defect (ASD) patient (99 NM, see Table 1). The patient was heterozygous for this mutation which would affect an arginine residue before the basic helix-loop-helix (bHLH) domain of HAND1. Furthermore, except for one positive case for dbSNP rs34402828 (c.468T>G, p.156S), no other sequence alterations were observed.

The *NKX2-5* and *HAND1* mutations described above have not been detected yet in our Leipzig collection of malformed hearts nor in at least 100 blood samples from unaffected individuals used as controls. They have not been reported as dbSNPs nor have been identified previously in association with CHD. Bioinformatics analysis by PolyPhen or SIFT for the NKX2-5 p.A119E and p.A119S mutations as well as the HAND1 p.R84L predicted a benign impact of those amino acid changes.

Search for mutations affecting binding domains or regions associated with cardiac malformations in other transcription factor genes did not identify sequence alterations except dbSNPs. For instance, we investigated 49 tissues for mutations of zinc fingers of *GATA4*, 49 tissues for mutations in amino acids affecting N-terminal (exons 2, 3) of *TBX5,* and 25 tissues (the first 17 patients, Table 1) for the whole gene *BMP4.*

### The p.A119E and p.A119S NKX2-5 mutants exhibited subtle yet significant functional defects in a yeast-based assay, while HAND1 p.R84L appeared as wild type like

We investigated the transactivation potential of the p.A119E-containing *NKX2-5* haplotype and the p.A119S mutation using a functional assay developed in *Saccharomyces cerevisiae* that was used successfully in dissecting clinical relevance of mutations in CHD [6]–[8]. The assay relied on the inducible expression in yeast of the entire human *NKX2-5* coding sequence fused with an acidic transactivation domain (TAD). In this assay, the NKX2-5 homeodomain is required for sequence-specific DNA binding at a chromosomally located luciferase reporter gene containing specific NK response elements, while the ectopic transactivation domain provide for efficient interaction with the yeast transcriptional machinery and transactivation [8]. The mutant p.A119E (c.356C>A) was found in *cis* with the synonymous coding variant c.543G>A (p.Q181) and the dbSNP c.63A>G (p.E21), as well as the dbSNP rs2277923 in the 3′UTR region, (see Fig. 1B). To examine the potential contribution of the two other sequence variants in the coding region on the functional properties of the p.A119E mutant, we also developed expression plasmids where the p.A119E was separated from c.543G>A sequence and the c.63A>G dbSNP.

**Figure 2:**
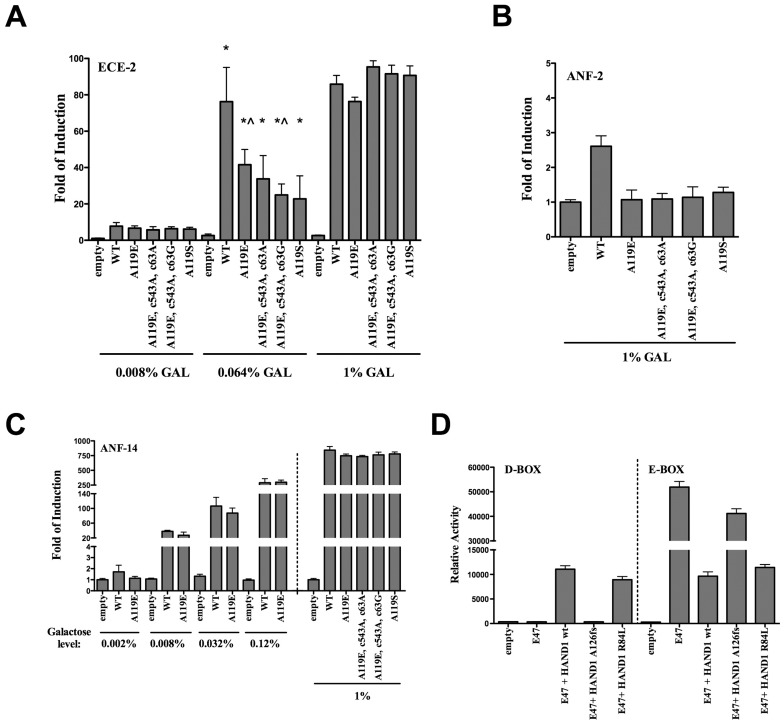
Transactivation potential of the p.A119E and p.A119S *NKX2-5* mutations. Presented in **A-C** is the average fold of luciferase reporter induction, relative to the activity measured at the lower galactose concentration in the absence of NKX2-5 proteins. Error bars correspond to the standard deviation of at least three biological repeats. Results were obtained with a reporter strain where the luciferase gene is controlled by a minimal promoter containing two repeats of the NK-RE derived from the ECE target gene (**A**), two repeats of the low-affinity NK-RE derived from the ANF target gene (**B**) or 14 repeats of the ANF NK-RE (**C**). The different NKX2-5 mutants and the galactose concentrations used to modulate expression are indicated. With the ECE reporter, the activity of p.A119E and p.A119S is significantly, albeit modestly reduced compared to wild type NKX2-5 (*  =  wt NKX2-5 compared to each mutant alleles; p<0.05; t-test). The haplotype found in patient #58LM [c.356A (p.A119E) + c. 543A (p.Q181) + c.63G (rs2277923)] further reduced transactivation (?  =  A119E mutant alone compared to patient #58LM haplotype; p<0.05; t-test). The reference (wild type) *NKX2-5* NM_004387.2 used here as control has the haplotype [c.356C + c. 543G + c.63A]. (**C**) The p.A119E mutation alone was tested with the highly responsive ANF-14 reporter strain using 4 different levels of protein induction obtained using the indicated concentrations of galactose in the medium. The complete panel of alleles was examined at high expression levels. (**D**) The impact of the HAND1 p.R84L mutation was examined using D-box and E-box reporter strain in co-expression experiments with E47–see text for details- The average relative light units normalized for the optical density of the cultures (OD_600nm_) and the standard deviations of 4 replicates are presented.

To measure the transactivation potential of the mutants, we used three different luciferase reporter strains that were previously constructed [8]. In particular, we chose the ECE2d reporter strain because it exhibited the highest discrimination among a group of NKX2-5 homeodomain mutants. This strain contains two repeats of the consensus NK-response element (RE) derived from the NKX2-5 downstream target gene endothelin-converting enzyme 1 [9]. We also used a strain containing two copies of NK-RE derived from the ANF target promoter, which resulted in weak responsiveness to NKX2-5, based on previous results and consistent with the presence of a mismatch in the RE [8]. We took advantage of the galactose inducible *GAL1,10* promoter to achieve variable expression of the NKX2-5 alleles which can be adjusted by the amount of galactose added to the medium, and compared the transactivation potential of the mutants and sequence variants relative to the wild type protein when expressed at different levels (Fig. 2A). Relative to wild type NKX2-5, the p.A119E and p.A119S mutants revealed a subtle, yet significant transactivation defect that was dependent on the level of expression and resulted at best in a 40% reduction in activity (0.064% galactose) in the ECE-RE strain. The combination of the p.A119E mutation with the c.543G>A and c.63A>G variants appeared to further slightly reduce the transactivation potential. The p.A119E-containing NKX2-5 haplotype as well as the separated mutations and p.A119S were however indistinguishable from the wild type at high expression levels (1% galactose) (Fig. 2A). Presumably the high expression compensated for the negative effects of the mutations. Using the weakly responsive ANF-RE strain at high expression levels (1% galactose), condition in which the wild type NKX protein resulted in ∼3 fold induction of the reporter, the p.A119E mutation alone or combined with the sequence variants, as well as p.A119S mutants appeared as loss of function (Fig. 2B).

The separated p.A119E mutation was also examined in a reporter strain containing 14 copies of the ANF-RE, which provides for high-level of induced transactivation. A defect was apparent also in this strain but only at low expression levels (Fig. 2C). All the mutant alleles were indistinguishable from the wt at high expression using this highly responsive strain (Fig. 2C).

We also examined the HAND1 p.R84L mutation identified in one ASD patient using the functional assays that we developed in yeast based on a pair of isogenic reporter strains, one containing the luciferase cDNA under control of a D-box RE, the other an E-box RE [7]. The assay exploits constitutive expression of the class A bHLH E47 protein that exhibits transactivation potential selectively towards the E-box reporter (Fig. 2D). Co-expression of wild type HAND1 severely inhibits E47 activity towards the E-box, but leads to transactivation from the D-box. The HAND1 p.R84L was slightly, if at all, less able to stimulates D-box together with E47 and was virtually wild type in inhibiting E47 on the E box. The previously studied HAND1 A126fs mutant [7] was included as control and behaved as a loss-of-function allele as expected.

**Figure 3:**
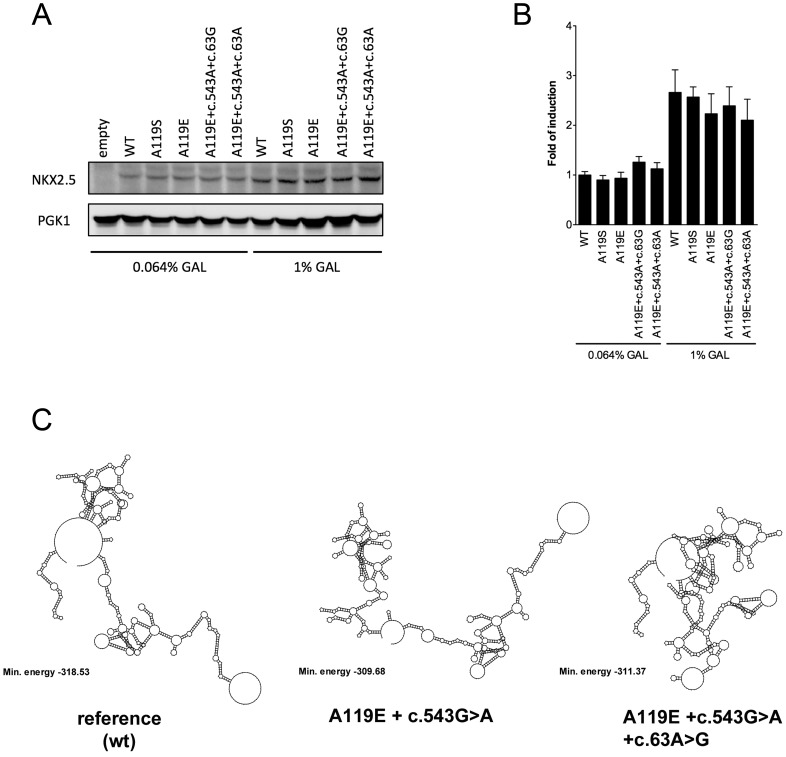
Western blotting at different expression levels and predictions of NKX2-5 RNA folding. (**A**) Yeast strain yLFM_ECE-2 harboring plasmid borne NKX2-5 alleles as indicated were grown to exponential phase at 0.064% as well 1% galactose and harvested after 24 hours. 100 µg of whole cell extract was subjected to gel electrophoresis through a 7.5% SDS polyacrylamide gel, transferred to a nitrocellulose membrane, and subjected to immunoblotting using antibodies against the ectopic transactivation domain present at the N-terminus of all the NKX2-5 proteins and against PGK1 used as reference. (**B**) Densitometric analysis of two independent Western blots experiments. Results are presented relative to the abundance of wt NKX2-5 measured from extracts obtained with yeast cells grown at 0.064% galactose. PGK1 was used as reference. The error bars represent the range of variation of the two independent experiments. (**C**) Predictions on mRNA secondary structure, as determined by the Vienna RNA folding procedure, showing differences in folding and minimum free energy between reference (wild type) and in the combination of variant alleles. The predicted minimum free energy values for the combined variants suggest less stable structures as compared to the reference. The 975-bp coding sequence of *NKX2-5* was used for the prediction.

Taken collectively, these results indicate that the p.A119E and p.A119S germline *NKX2-5* mutations have a negative impact on sequence-specific transactivation (Fig. 2A). Western Blot analysis was conducted with protein extracts prepared from cultures grown in 0.064% and 1% galactose. Protein amounts could not be reliably examined at low expression expression levels (0.008% galactose). Similar steady-state protein levels for all alleles were apparent at both galactose concentrations tested (Fig. 3A). From the quantification of the WB immunoblot (Fig. 3B), we can conclude that there was less than three fold difference in protein expression between the two galactose concentrations and that p.A119E and p.A119S mutants did not affect the stability of the protein.

Using the coding sequence of *NKX2-5* and the Vienna RNA folding algorithm, we predicted differences in mRNA secondary structure between the reference (wild type) and in the p.A119E-containing CHD haplotype suggesting that changes in mRNA stability and folding might contribute to the functional impact (Fig. 3C and Fig. S1). Predictions were obtained for the entire coding sequence as well as the entire mRNA transcript. While the p.A119E mutation was predicted to have a subtle impact on the stability of the mRNA, the dbSNP c.63A>G and the c.543G>A synonymous variant were predicted to impact on its folding. Given that the yeast-functional assay does not utilize the entire NKX2-5 mRNA, but a chimeric cDNA lacking UTRs, these observations, although speculative, may suggest that the haplotype structure could have an impact *in vivo* also on NKX2-5 mRNA fate and translation efficiency.

## Discussion

Previously, we conducted genetic studies in different transcription factor genes such as *NKX2-5, GATA4*, *TBX5*, *HEY2* and *HAND1* using a morphologically well-characterized heart collection with complex malformations [10]. These studies on the Leipzig heart collection suggest possible role of somatic mutations in CHD, in which mutations in affected tissues were generally absent in unaffected tissues of same malformed hearts, or normal hearts from the same collection. In the present work, we investigated genomic DNA isolated from discarded cardiac biopsies of CHD patients undergoing heart surgery. In contrast to the Leipzig malformed hearts, where there was sizable amount of material to be studied in both affected and unaffected regions, the origin and size of the biopsies could not be dictated. Clearly, the tissues were minute and mostly not within malformations. Nevertheless, examining cardiac tissues, when available, may be advantageous as differences can occur between blood, diseased and normal tissue samples in an individual as recently reported for abdominal aortic aneurysm [11].

In the present study, we examined 49 biopsies from 28 patients and except for three patients harboring *NKX2-5* or *HAND1* mutations, no other mutations were identified even after further analysis of specific regions of the *GATA4*, *TBX5* and *BMP4* corresponding to key functional domains of the respective proteins. Further analysis with DNA extracted from blood samples of the patients and their parents revealed the germline origin of the identified mutations. No somatic mutations were found in the present material. Reasons for non-detection may be due to analysis of the wrong material (as already discussed above), amount of tissue, sensitivity of direct DNA sequencing, or true absence owing to other causes of CHD, be it genetic or epigenetic. The sensitivity of our direct sequencing was determined to be about 20% for a mixture of variant allele over total DNA. Specifically, PCR fragments homozygous for *NKX2-5* allele dbSNP c. 63A or G were mixed with varying proportions, resulting in heterozygous condition with decreasing quantity of allele G, and analyzed by sequencing (not shown).

The two *NKX2-5* nonsynonymous germline mutations identified in the present study led to single amino acid changes at the same position of the protein (p. A119), and were associated with different cardiac disease phenotypes. For instance, p.A119E was found in a patient with AVSD, while p.A119S was found in a patient with HLHS, who succumbed to the disease. Whether the identified *NKX2-5* germline mutations are clinically relevant, remains to be determined. Firstly, the unaffected parents displayed the mutations (see Fig. 1B, C), but had a normal heart. Similarly, p.A119S was also carried by the patient's unaffected mother. Secondly, p.A119S was previously detected in a patient with ectopic thyroid but no documented CHD [12]. Besides p.A119S, two other *NKX2-5* mutations [i.e. c.73C>T (p.R25C) and c.482G>C (p.R161P)] were found in thyroid dysgenesis (TD), in patients with or without cardiac anomalies, to suggest *NKX2-5* mutations to have variable penetrance and a broader impact in organogenesis and pathophysiology.

Recently, the role of p.A119S germline mutation in CHD and TD was challenged [13]. In this study only 2 out of 303 patients with ASD and 38 study subjects from families with CHD were carrier of the variant allele. The authors therefore concluded that genetic testing for *NKX2-5* mutations in TD is not warranted. It must be noted, however, that the detection frequency of NKX2–5 mutations in sporadic cases of CHD is about 2%, and in several studies none was found. As summarized in our recent update on this hypermutable homeodomain protein and its role in human CHD, most of the identified NKX2–5 mutations are unique, and diverse cardiac malformations have been associated with NKX2–5 mutations [14]. Indeed, the same CHD phenotypes have been exhibited by patients with different NKX2–5 mutations, or the same NKX2–5 mutation gave different CHD phenotypes with regard to severity even within families. At least 41 different NKX2–5 germline mutations, most of which lead to amino acid change have been reported. However, only 5 of 41 (12%) have been reported more than once in unrelated individuals, with c.73C4T (p.R25C) being frequently detected but p.R25C was also found in unaffected relatives and controls; hence, its pathogenicity remains unclear.

In the study of Engelen et al. [13] the functional consequence of the p.A119S variant in a rat heart derived H10 and in HeLa cells was investigated and no difference between wildtype NKX2-5 and p.A119S NKX2-5 in an activation of the investigated promoters was observed. However, the findings of Engelen et al. [13] differ from those reported by Dentice et al. [12] who likewise investigated the transcriptional properties of WT and the p.A119S NKX2–5 mutant in HeLa cells. In their study the mutant p.A119S NKX2-5 was able to activate the reporter gene in a dose-dependent manner, but the activity was reduced at all tested concentrations in all assays performed when compared with WT. Moreover, this effect was not related to a reduction in the mutant protein concentration, as confirmed by Western blot analysis and this agrees with the findings of the present study (see Fig. 3A). Furthermore, co-expression of WT NKX2-5 with the same amount of mutant protein resulted in a reduction of luciferase activity, suggesting a dominant-negative effect of the mutant protein [12].

To explore potential clinical significance, functional analysis of p.A119E and p.A119S mutants was carried out in a yeast-based assay. Interestingly with p.A119E, the presence of a synonymous variant c.543G>A (p.Q181), and especially in the combination with the synonymous dbSNP c.63A>G (p.E21) in *cis*, as found in the patients' haplotype, led to further reduction of transactivation activities.

The combination c.543G>A and c.63A>G was also detected in a patient with a secundum ASD suggesting their potential role in CHD [15].

Indeed, synonymous variants may not be silent as assumed to be as they could affect protein expression and function [16]. For instance, synonymous mutations in human dopamine receptor D2 (*DRD2*) impaired mRNA stability and synthesis of the receptor [17]. Moreover, there is emerging knowledge about the occurrence of multiple mutations in the same gene or different genes being an indication of disease severity in genetic cardiovascular disease [18]. Here we show a family in which concomitant transmission of nonsynonymous and synonymous variant alleles impacted transcriptional activity of NKX2-5. While the missense changes appear sufficient to negatively impact on NKX2-5 transactivation potential, the synonymous changes may further contribute to the phenotype, possibly acting at a post-transcriptional level. It is important to note that while sensitive to subtle functional defects caused by coding mutations, the yeast-based assay we used, that is based on ectopically expressed cDNA, may underestimate the post-transcriptional effect of coding sequence changes.

Altogether, we identified only 3 of 28 positive cases, and while *NKX2-5* and *HAND1* mutations were observed in septation defects, mutations that affect the same amino acid of NKX2-5 were associated with different disease phenotypes, i.e. AVSD and HLHS. No germline mutation was found associated with HLHS in *HAND1*, a candidate gene for this CHD type based on results from animal models [19], [20] and our investigations with the Leipzig heart collection [6]. These observations indicate that CHD is a complex disease and that mutations in different genes may lead to the same disease phenotype, or conversely mutations in the same gene resulting in different disease phenotypes. Furthermore, functional studies showed that the presence of additional *cis* synonymous variants including a dbSNP transmitted from an unaffected parent reduced further the transcriptional activity of the *NKX2-5* mutation. Lastly, NKX2-5 displays hypermutability but there is lack of genotype-phenotype correlation of *NKX2-5* mutations as recently reviewed by us [14]. For one, genetic background can modify the phenotypic expression of single gene mutations in mice and humans [21] and modifier genes have been shown to affect NKX2–5 mutations in the pathogenesis of CHD [22], [23]. There is also the possible influence of synonymous (silent) mutations and dbSNPs affecting protein expression and function as shown in the present study and by other investigators [16]. Such silent variants and dbSNPs eventually serve as risk factors for CHD. Furthermore, buffering that can result from compensation by a normally functioning second allele or a duplicated gene or a pathway that maintains residual function may also explain reduced penetrance of mutations in sporadic cases of CHD (see review [24]). These authors proposed that the genetic architecture of sporadic CHD likely includes accumulation of rare nonsynonymous variants in cardiac developmental genes leading to mutational loading of cardiac developmental networks, copy number variation in cardiac developmental genes, and common variants that may not be obviously linked to cardiac development but may alter genetic buffering pathways. Moreover, germline mutations may not be compatible with survival of the developing embryo because many of the genes that control cardiac development also play critical roles in the development of other organ systems, and in any case, would not likely cause CHD only [25].

Summing up, an interplay of genetic, epigenetic and environmental effects need to be considered in disease pathogenesis. Thus, a simple genetic analysis may not reveal causation.

## Supporting Information

Figure S1 **Predictions of the NKX2-5 mRNA secondary structure.** Taking advantage of the RNAfold web server (http://rna.tbi.univie.ac.at/cgi-bin/RNAfold.cgi) the mRNA secondary structure was developed for the entire NKX2-5 transcript (1585nt), complementing the analysis on the coding sequence presented in Figure 3B. The reference (wild type), the c.356C>A mutation, resulting in the p.A119E amino acid change as well as c.543G>A variant and the c.63A>G dbSNP present in the p.A119E-containing patient's haplotype were compared. The predicted minimum free energy (kcal/mol) is indicated. The c.356C>A mutation is not predicted to affect the mRNA secondary structure, but to slightly weaken a long stem and to slightly reduce the minimum free energy of the folded mRNA. Both the c.543G>A variant and the c.63A>G dbSNP are instead predicted to alter the folding of the mRNA, with no significant impact on stability.
(TIF)
