# Supplementary figures and images for: Transcriptional Defect of an Inherited NKX2-5 Haplotype Comprising a SNP, a Nonsynonymous and a Synonymous Mutation, Associated with Human Congenital Heart Disease
Source: PLoS One. 2013 Dec 20;8(12):e83295. doi: 10.1371/journal.pone.0083295 (PMC3869772; doi:10.1371/journal.pone.0083295)

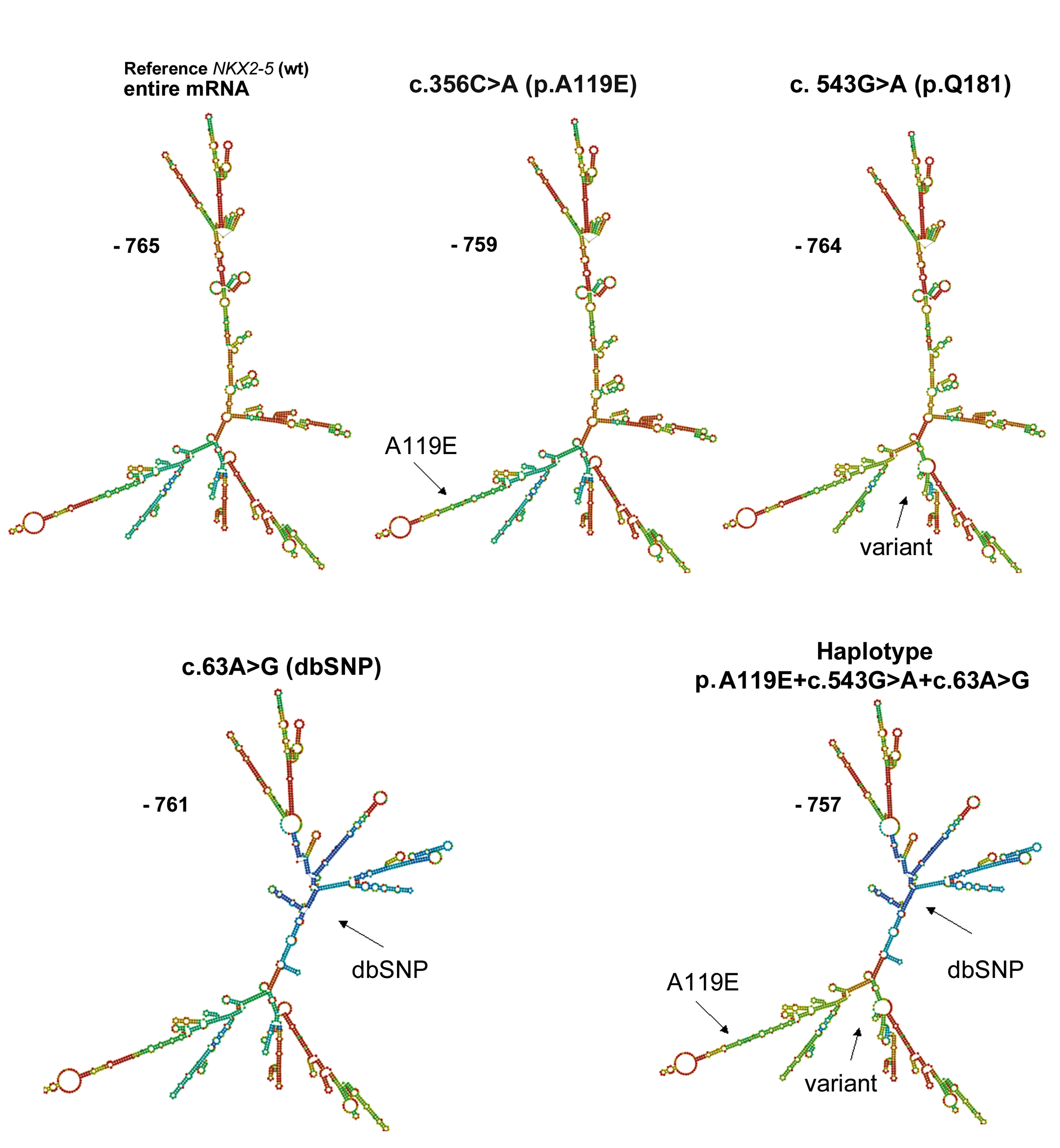

Supplement: Figure S1 — Predictions of the NKX2-5 mRNA secondary structure. Taking advantage of the RNAfold web server (http://rna.tbi.univie.ac.at/cgi-bin/RNAfold.cgi) the mRNA secondary structure was developed for the entire NKX2-5 transcript (1585nt), complementing the analysis on the coding sequence presented in Figure 3B. The reference (wild type), the c.356C>A mutation, resulting in the p.A119E amino acid change as well as c.543G>A variant and the c.63A>G dbSNP present in the p.A119E-containing patient's haplotype were compared. The predicted minimum free energy (kcal/mol) is indicated. The c.356C>A mutation is not predicted to affect the mRNA secondary structure, but to slightly weaken a long stem and to slightly reduce the minimum free energy of the folded mRNA. Both the c.543G>A variant and the c.63A>G dbSNP are instead predicted to alter the folding of the mRNA, with no significant impact on stability. (TIF) [file pone.0083295.s001.tif]
